# Spatial access to primary care providers and colorectal cancer‐specific survival in Cook County, Illinois

**DOI:** 10.1002/cam4.2957

**Published:** 2020-03-04

**Authors:** Vincent L. Freeman, Keith B. Naylor, Emma E. Boylan, Benjamin J. Booth, Oksana Pugach, Richard E. Barrett, Richard T. Campbell, Sara L. McLafferty

**Affiliations:** ^1^ Division of Epidemiology and Biostatistics School of Public Health University of Illinois at Chicago Chicago IL USA; ^2^ University of Illinois Cancer Center University of Illinois Hospital and Health Sciences System Chicago IL USA; ^3^ Division of Gastroenterology & Hepatology College of Medicine University of Illinois at Chicago Chicago IL USA; ^4^ Office of Community Health Systems Washington State Department of Health Olympia WA USA; ^5^ Institute of Health Research and Policy School of Public Health University of Illinois at Chicago Chicago IL USA; ^6^ Department of Sociology College of Liberal and Sciences University of Illinois at Chicago Chicago IL USA; ^7^ Department of Geography and Geographic Information Science University of Illinois at Urbana‐Champaign Urbana IL USA

**Keywords:** colorectal cancer, primary care, prognosis, residence characteristics

## Abstract

**Background:**

Spatial access to primary care has been associated with late‐stage and fatal breast cancer, but less is known about its relation to outcomes of other screening‐preventable cancers such as colorectal cancer. This population‐based retrospective cohort study examined whether spatial access to primary care providers associates with colorectal cancer‐specific survival.

**Methods:**

Approximately 26 600 incident colorectal cancers diagnosed between 2000 and 2008 in adults residing in Cook County, Illinois were identified through the state cancer registry and georeferenced to the census tract of residence at diagnosis. An enhanced two‐step floating catchment area method measured tract‐level access to primary care physicians (PCPs) in the year of diagnosis using practice locations obtained from the American Medical Association. Vital status and underlying cause of death were determined using the National Death Index. Fine‐Gray proportional subdistribution hazard models analyzed the association between tract‐level PCP access scores and colorectal cancer‐specific survival after accounting for tract‐level socioeconomic status, case demographics, tumor characteristics, and other factors.

**Results:**

Increased tract‐level access to PCPs was associated with a lower risk of death from colorectal cancer (hazard ratio [HR], 95% confidence interval [CI]) = 0.87 [0.79, 0.96], *P* = .008, highest vs lowest quintile), especially among persons diagnosed with regional‐stage tumors (HR, 95% CI = 0.80 [0.69, 0.93], *P* = .004, highest vs lowest quintile).

**Conclusions:**

Spatial access to primary care providers is a predictor of colorectal cancer‐specific survival in Cook County, Illinois. Future research is needed to determine which areas within the cancer care continuum are most affected by spatial accessibility to primary care such as referral for screening, accessibility of screening and diagnostic testing, referral for treatment, and access to appropriate survivorship‐related care.

## Graphical abstract

In this population‐based retrospective cohort study of approximately 26 600 incident colorectal cancers diagnosed in Cook County, IL between 2000 and 2008, spatial access to primary care was associated with colorectal cancer‐specific survival independent of neighborhood socioeconomic status, tumor stage, and other factors. To elucidate the underlying mechanisms, future research is needed to determine which areas within the cancer care continuum are most affected by spatial accessibility to primary care.

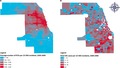

## 1 INTRODUCTION

Colorectal cancer (CRC) continues to be the second leading cause of death from cancer in the United States, with 145 600 new cases and 51 020 deaths expected in 2019.^1^ Despite strong hereditary components, most cases of colorectal cancer are sporadic and largely attributable to a spectrum of modifiable interrelated factors.^2^, ^3^, ^4^ Health behaviors such as low physical activity, alcohol consumption, and poor diet quality (eg, low consumption of fiber and whole grain, high consumption of red and processed meats) are established risk factors.^5^ The removal of precursor lesions found during guideline‐concordant screening and the detection of CRC at an early stage significantly reduces incidence and CRC‐related mortality.^6^ Moreover, there is mounting evidence that characteristics of the built environment such as land use, transportation infrastructure, and access to preventive services can influence CRC prevention and control through their effects on health behaviors and health services utilization.^7^ Thus, colorectal cancer is one of the most avoidable causes of death in the general US population.

Among persons with CRC, tumor stage at diagnosis and receipt of evidence‐based treatment are the major determinants of CRC‐specific survival. However, there is increasing evidence that factors not directly related to the tumor or its treatment are also prognostic. For example, lifestyle before and after diagnosis associates with disease‐specific survival, with the most consistent findings being increased survival with physical activity and decreased survival with red and processed meat consumption.^8^ Residential factors have also been associated with variation in CRC outcomes, including whether the patient lives in an urban vs a rural setting and the socioeconomic context of the patient's community or other geography of residence.^9^, ^10^

More recently, there has been increasing recognition of the effect of spatial accessibility to healthcare on cancer survival.^11^, ^12^ Spatial accessibility refers to the availability of a service moderated by distance and, in a healthcare context, is interpreted as a measure of potential access to various components of the healthcare system.^13^ These components are often not distributed equitably across the United States; some areas have an excess of healthcare providers and services while other struggle to meet basic healthcare needs.^14^ A study in Texas examined CRC‐specific survival in relation to census tract‐level access to oncologists using an enhanced two‐step floating catching area method to measure spatial accessibility.^15^, ^16^ After accounting for patient, tumor, and area‐level sociodemographic factors, CRC‐specific survival was positively associated spatial access to oncologists in rural areas but not in urban areas.

Primary care is a necessary component of effective healthcare systems and for maintaining population health.^17^, ^18^ Additionally, primary care providers can play critical roles across the cancer continuum, from primary prevention through to survivorship.^19^, ^20^ Spatial access to primary care has been associated with late‐stage and fatal breast cancer in a small number of studies.^21^, ^22^, ^23^ However, even less is known about its relation to outcomes of other screening‐preventable cancers such as those of the colon and rectum. Therefore, the objective of this population‐based retrospective cohort study was to examine whether spatial access to primary care providers is associated with colorectal cancer‐specific survival. We hypothesized that increased spatial access to primary care providers (PCPs) would be associated with increased survival, even after accounting for tumor stage at diagnosis, area‐level socioeconomic status, competing risk of death from other causes, and other factors.

The setting for this study was Cook County, Illinois (USA). Cook County is the second most populous county in the United States, has a strong urban/suburban divide, and is racially, ethnically, and socioeconomically diverse.^24^ Chicago is the dominant municipality and a major hub of medical care and training. The county also has one of the highest concentrations of PCPs in Illinois, accounting for nearly half of all PCPs practicing in the state.^25^ Lastly, over 40% of CRC‐related deaths in the state occur in Cook County, IL.^26^

## 2 METHODS

### 2.1 CRC survival data

*Study Population*—After obtaining Institutional Review Board Approvals, we used the Illinois State Cancer Registry (ISCR) to identify and collect relevant data on all incident cases of colorectal cancer diagnosed between January 1, 2000 and December 31, 2008 in persons residing in Cook County, IL. ISCR is the only source of population‐based cancer incidence data for the state. Newly diagnosed cancer cases in Illinois residents are reported to ISCR through mandated reporting by healthcare facilities where the cancer is diagnosed and/or treated. Central cancer registries and facilities in several states (including all states bordering Illinois) also report data to ISCR on Illinois residents diagnosed and/or treated for cancer in their states. Approximately 5.4% of cases come from out‐of‐state, and the completeness of ascertainment is estimated at 98%.^27^ The North American Association of Central Cancer Registries (NAACCR) has awarded ISCR the highest level of certification (“Gold Certification”) every year since 1995.^28^

**FIGURE 1:**
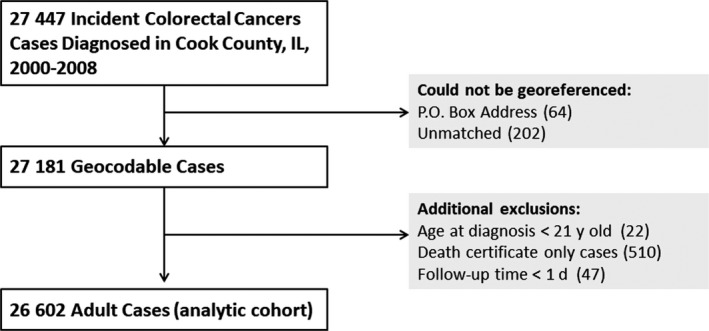
Selection of the final analytic cohort. Cases diagnosed in the Veterans Health Administration healthcare system were not available for analysis. Total number of cases excluded was 845 (3.1%)

*Case Definition*—All cancers with a primary site of the colon or rectum were extracted from ISCR using the International Classification of Diseases for Oncology 3rd edition (ICD‐O‐3) codes C18, C19, C20, and C26.0,^29^ consistent with the US SEER Program's Site Recode definition of colorectal cancer (http://seer.cancer.gov/siterecode/). Cases excluded by the state tumor registry prior to releasing the data to the study's investigators were anus, anal canal, and anorectum cancers (ICD‐O‐3 code C21) and tumors with the following morphologies: hematopoietic malignancies, mesotheliomas, and sarcomas. Also, statutory restrictions prevented release of information on incident cases diagnosed in the Veterans Health Administration system. As a result, 27 447 cases were available to the study for analysis. A flowchart summarizing the selection of the final analytic cohort is shown in Figure 1.

*Georeferencing of Cases*—We used ArcGIS, version 10.1 (Environmental Service and Research Institute [ESRI], Inc) to geocode case mailing address at the time of diagnosis.^30^ Address range feature data for Cook County, Illinois from 2012 were obtained from the US Census Bureau TIGER/Line portal and were used as the address reference file.^31^ The minimum automatic match score was set to 73%, resulting in 26 672 (97%) matches, 145 (0.5%) ties, and 630 (2%) unmatched addresses. Ties were assigned to their initial match address, and all PO boxes (64) were left unmatched. Manual review of the remaining unmatched records resulted in 364 additional matches, bringing the total matches to 27 181 (99%).

*Vital Status, Underlying Cause of Death, and Survival Time*—Follow‐up of the cohort was censored as of January 31, 2012. Vital status and underlying cause of death of decedents were ascertained using the National Death Index Plus, and underlying cause of death coded based on the International Classification of Diseases, 10th Revision (ICD‐10).^32^, ^33^ Survival time (in days) was computed for each case beginning with the CRC diagnosis date and ending on either the end‐of‐follow date, date of death from colorectal cancer (ICD‐10 codes C18‐C21), or date of death from another underlying cause, whichever came first.

*Additional Exclusions*—Death certificate‐only cases (510), cases less than 21 years old at time of diagnosis (22), and those with survival time less one day were excluded, leaving 26 602 cases for the final analytic cohort. Compared to the final analytic cohort, excluded cases (n = 845, 3.1%) were significantly more likely to be non‐Hispanic black, younger than 45 years or 75 years or older, of unknown tumor stage, and to have died of colorectal cancer by the end of the follow‐up period.

### 2.2 Measuring spatial access to PCPs

*Georeferencing of PCP Locations*—Our methods for ascertaining and georeferencing PCP locations have been described elsewhere.^34^ Briefly, physician practice and location data were obtained for Cook County, IL and neighboring counties from the American Medical Association's Physician Masterfile (https://www.ama-assn.org/practice-management/masterfile), with data available for 2000 and for each year from 2003 to 2008. We defined PCPs as physicians whose primary specialty was general internal medicine, family practice, or general practice. Physicians reported as dead or inactive were excluded, as well as those who did not deal directly with patient care, such as those who were primarily researchers or administrators. Automatic geocoding was performed using the North America Geocode Service in ArcGIS, version 10.0 (ESRI, Inc). Addresses left unmatched were geocoded manually. Approximately 6% of physicians did not report an office address and therefore were geocoded to their preferred mailing address location. Some physicians ultimately could not be geocoded due to missing or invalid addresses such as PO box addresses (range, 0.8% to 1.4% across years). The annual number of geocoded PCPs ranged from 5783 in 2000 to 6219 in 2008.

*Measuring Spatial Access PCPs*—Spatial access to PCPs was measured based on census‐tract boundaries using an enhanced two‐step floating catchment area (E2SFCA) method introduced by Luo and Qi that accounts for distance decay.^16^ A 2‐step floating catchment area (2SFCA) method involves two interrelated steps.^35^ The first step begins with each physician location (j) and determines the census tracts (k) that are within a threshold distance or travel time (d_0_). From this, a physician‐to‐population ratio R*_j_* within the catchment area of each physician is calculated (Equation 1), where *P_k_* is the population of census tract *k*, *S_j_* is the number of physicians at location *j*, and *d_kj_* is the travel time between *k* and *j*.

(1) $$R_{j}=\frac{S_{j}}{\sum_{k\in \left\{d_{\mathrm{kj}}\leq d_{0}\right\}}P_{k}}$$

The second step starts with each population area (eg, a census tract) *i*, and sums the physician‐to‐population ratios from equation 1 for all physician locations within the travel time threshold of *k*. This estimates the spatial accessibility for census tract *i* (A*_i_*) (Equation 2).

(2) $$A_{i}=\sum_{j\in \left\{d_{\mathrm{ij}}\leq d_{0}\right\}}R_{j}=\sum_{j\in \left\{d_{\mathrm{ij}}\leq d_{0}\right\}}\left(\frac{S_{j}}{\sum_{k\in \left\{d_{\mathrm{kj}}\leq d_{0}\right\}}P_{k}}\right)$$

Thus, this method determines the total physician‐to‐population ratio within a particular travel time for population residing in census tract *i*.

The enhanced method extends this approach to incorporate declining levels of spatial accessibility with increasing travel time or distance. In each step, values are weighted according to prespecified distance decay weights that decrease as travel time increases. A common approach is to create a series of travel time zones (eg, 0‐10 minutes, 10‐20 minutes) and to assign a constant weight (W*_r_*) to all values within the zone. In step 1 of the method, these distance weights are assigned to the population values:

(3) $$R_{j}=\frac{S_{j}}{\sum_{j\in \left\{d_{\mathrm{kj}}\in D_{r}\right\}}P_{k}W_{r}}$$

S*_j_* represents the number of PCPs at *j*, P*_k_* denotes the population size of any census tract *k* within the catchment, d*_kj_* represents the shortest travel time between *j* and *k*, D*_r_* denotes the *r* ^th^ travel time zone

In the second step, the E2SFCA shifts to each population location and computes the weighted sum of physician‐to‐population ratios within the fixed travel time catchment of that location *i* (Equation 4):

(4) $$A_{i}^{F}=\sum_{l\in \left\{d_{\mathrm{il}}\in D_{r}\right\}}R_{l}W_{r}$$

where R*_l_* is the supply‐to‐demand ratio of any PCP location *l* inside the catchment and d*_il_,* the travel time between *l* and *i*. The resulting values represent the travel‐time‐weighted physician‐to‐population ratios for each population zone, known as the spatial access “score” of *i*

Our implementation of the E2SFCA followed Luo and Qi,^16^ with the spatial access scores calculated using the Network Analyst Extension in ArcGIS, version 10 (ESRI, Inc, Redlands, CA, USA). We define the maximum travel time (catchment size) as 30 minutes. Within this, three travel time zones are created (0‐10, 10‐20, and 20‐30 minutes), with corresponding weights of 1, 0.68, and 0.22, respectively.^16^ Travel times by car are estimated from each census tract to each physician location based on the road network and typical travel speeds. We included physician practice locations outside of the Cook County boundary to account for potential access to PCPs in neighboring Illinois counties. To account for the spatial variation in populations within Cook County census tracts, a population‐weighted centroid was calculated for each census tract based on the populations of census blocks within the tract.^36^ These centroids were used in the estimation of travel times.

In summary, we used the E2SFCA method to calculate a spatial access “score” (ie, travel‐time‐weighted physician‐to‐population ratio) for each census tract in Cook County, IL (n ~ 1350) for each of the nine diagnosis years of the cohort, 2000 through 2008. Since AMA Physician Masterfile data on PCP practice locations in Cook County were not available for 2001 and 2002, we used census tract‐level PCP access scores from 2000 and 2003 to estimate the corresponding scores in 2001 and 2002 using linear interpolation.

### 2.3 Covariates

*Census Tract‐Level Concentrated Disadvantage*—Concentrated disadvantage (CD) is a multidimensional area‐based indicator of socioeconomic status (SES) derived by Browning and Cagney and, in various formulations, has been associated with self‐rated health and cancer‐specific survival in Cook County.^37^, ^38^, ^39^ In this study, we used the simple sum of %poverty + %unemployed + %female‐headed households + (100 − %college graduate).^39^ CD scores were calculated for each Cook County census tract in the year 2000 using information from the 2000 US decennial census and in years 2001‐2008 using the Neighborhood Change Database.^40^, ^41^

*Case Demographics*—We created the following eight race/ethnicity groups based on the NAACCR Hispanic Identification Algorithm (NHIA) and Asian/Pacific Islander Identification Algorithm (NAPIIA) categories assigned to each case by the state cancer registry: white, non‐Hispanic; black, non‐Hispanic; Hispanic; Asian; Pacific Islander; Asian‐Indian/Pakistani; American Indian, Alaskan Native or Other; or unknown. Sex was categorized as either male or female, and age was based on integer age at the time of the cancer diagnosis.

*Tumor Characteristics*—ICD‐O‐3 topography codes were used to define four anatomic subsite (“primary location”) groupings: proximal colon (C18.0, C18.2, C18.4, C18.5), distal colon (C18.6, C18.7), rectum (C19.9, C20.9), and “large intestine and not otherwise specified (NOS)” (C18.8‐C18.9, C26.0, C18.1).^29^ Stage at diagnosis was based on the SEER Summary Stage classification of either in‐situ, localized, regional (by direct extension, to lymph nodes, or NOS), distant, or unknown.^42^ Unknown stage cases were further subdivided into “missing” vs “deferred or refused” due to their distinct survival distributions.

*Reporting Facility Characteristics and Other Variables*—Reporting facilities were characterized with respect to the following: (a) safety‐net designation at the time of reporting (yes/no); (b) academic/university‐based as defined by the American Association of Medical Colleges (1997) (yes/no); (c) case volume defined as the number of CRC cases reported from Cook County and grouped into “High” = 75 to 149 per year (50%), “Medium” = 30 to <75 per year (35%), or “Low” <1 to <30 per year (13%), based on natural breaks in the distribution, or “not available” (~2%). Other variables were location of residence within Cook County at diagnosis (Chicago vs suburbs) and diagnosis year.

### 2.4 Data analysis

All analyses were performed using the statistical package SAS version 9.4 (SAS Institute, Inc) and ArcMap 10.7.1 (ESRI, Inc).

*Descriptive Analyses*—We used “FREQ” procedure in SAS to summarize the distribution of baseline characteristics and subsequent causes of death and Pearson *χ* ^2^ tests were used to compare selected characteristics of the analytic cohort to excluded cases. The “STDRATE” procedure was used to calculate CRC‐specific cumulative mortality rates during the follow‐up period at the census tract level using 2000 Census geography and seven age strata (<5, 5‐14, 15‐34, 35‐44, 45‐54, 55‐64, ≥65 years) and age‐standardized to the 2000 US population. We chose to map the cumulative number of fatal CRC cases per 10 000 residents because small number issues at the census tract level prevented us from mapping other indirect measures of CRC‐specific survival. We calculated the mid‐3‐year period (2003, 2004, and 2005) average PCP access score (PCPs per 10 000 residents) for each Cook County census tract. Tract‐level scores and rates were then joined to Census 2000 geography for Cook County, IL using ArcMap 10.7.1. to create two maps which compared the spatial distribution of mid‐period PCP access scores (quintiles) and corresponding CRC‐specific cumulative mortality rates (quintiles).

*Survival Analyses—*We used the "PHREG" procedure in SAS to analyze the association between census tract‐level PCP access scores and colorectal cancer‐specific survival based on a competing risks regression framework using Fine‐Gray proportional subdistribution hazard models.^43^, ^44^ The models incorporated tract‐level PCP access score (quintiles) in year of diagnosis (model 1), followed by serial adjustments for census tract‐level concentrated disadvantage (quintiles) in year of diagnosis (model 2); case demographics—race/ethnicity (non‐Hispanic white, non‐Hispanic black, Hispanic, Asian, Other), sex, age at diagnosis (model 3); tumor characteristics—primary anatomic subsite and stage (model 4); and reporting facility characteristics—safety‐net designated (yes/no), academic/university‐based (yes/no), and Cook County case volume (high, medium, low, or not available) (model 5), with statistical significance based on the Wald *χ* ^2^ test. The "COVSANDWICH" option was included to account for the nesting of cases by reporting facility. In this option, the standard errors for the model parameters are computed using the robust "sandwich" variance estimator of Wei and colleagues.^45^ We repeated the analysis to account for the nesting of cases by census tract, but results were virtually identical to those without the sandwich variance estimate. The analysis was further stratified by tumor stage. We checked the proportional subdistribution hazards assumption of all models by generating Schoenfield‐type residuals and plotting them against time using the “LOESS” procedure.^46^

## 3 RESULTS

Table 1 summarizes selected baseline characteristics and mortality outcomes of the CRC cohort. Cases were predominantly either non‐Hispanic white, non‐Hispanic black, or Hispanic (96%), age 65 years or older (66%), and slightly more likely to be female (51%).

**TABLE 1:** Characteristics of the study population

Characteristic		No.	%	
Sample size		26 602	100	
Race	White, non‐Hispanic	16 866	63.4	
	Black, non‐Hispanic	6934	26.1	
	Hispanic	1708	6.4	
	Asian	487	1.8	
	Pacific Islander	215	0.8	
	Asian‐Indian/Pakistani	136	0.5	
	American Indian, Alaskan Native or Other	77	0.3	
	Unknown	179	0.7	
Sex	Female	13 578	51.0	
	Male	13 024	49.0	
Age at Diagnosis, y	< 45	1096	4.1	
	45 to 54	2971	11.2	
	55 to 64	5098	19.2	
	65 to 74	6947	26.1	
	>75	10 490	39.4	
Primary Site	Proximal Colon	10 957	41.2	
	Distal Colon	6816	25.6	
	Colorectal Other & NOS	1522	5.7	
	Rectum	7307	27.5	
Stage	In Situ	2255	8.5	
	Local	9075	34.1	
	Regional	9056	34.0	
	Distant	4827	18.2	
	Unknown—Stage information missing	572	2.2	
	Unknown—Staging deferred or refused	817	3.1	
Median follow‐up time				45.7 mos.
Deaths	All	13 828	52.0	
	Due to colorectal cancer	8004	30.1	
	Due to other causes^a^	5824	21.9	
			

Note

Abbreviations: NOS, not otherwise specified.

See Table S3 for a comparison of the study population to excluded cases with respect to baseline characteristics, vital status, and causes of death.

^a^ Cardiovascular or diabetes‐related (n = 2196); other cancer (n = 1606); genito‐urinary, pulmonary, hematologic, or other gastro‐intestinal (n = 1057); infectious disease (n = 237); other (n = 728).

Consistent with national trends at that time,^47^ the most common anatomic subsite was proximal colon (41.2%), followed distal colon and rectum (25.6% and 27.5%, respectively), and the most common tumor stages were local and regional (approximately 34% each) followed by distant (18.2%) with approximately 5% classified as “unknown stage.” By the end of the follow‐up period, over half of the patients had died, with death due to CRC as the most common (58%) underlying cause.

**FIGURE 2:**
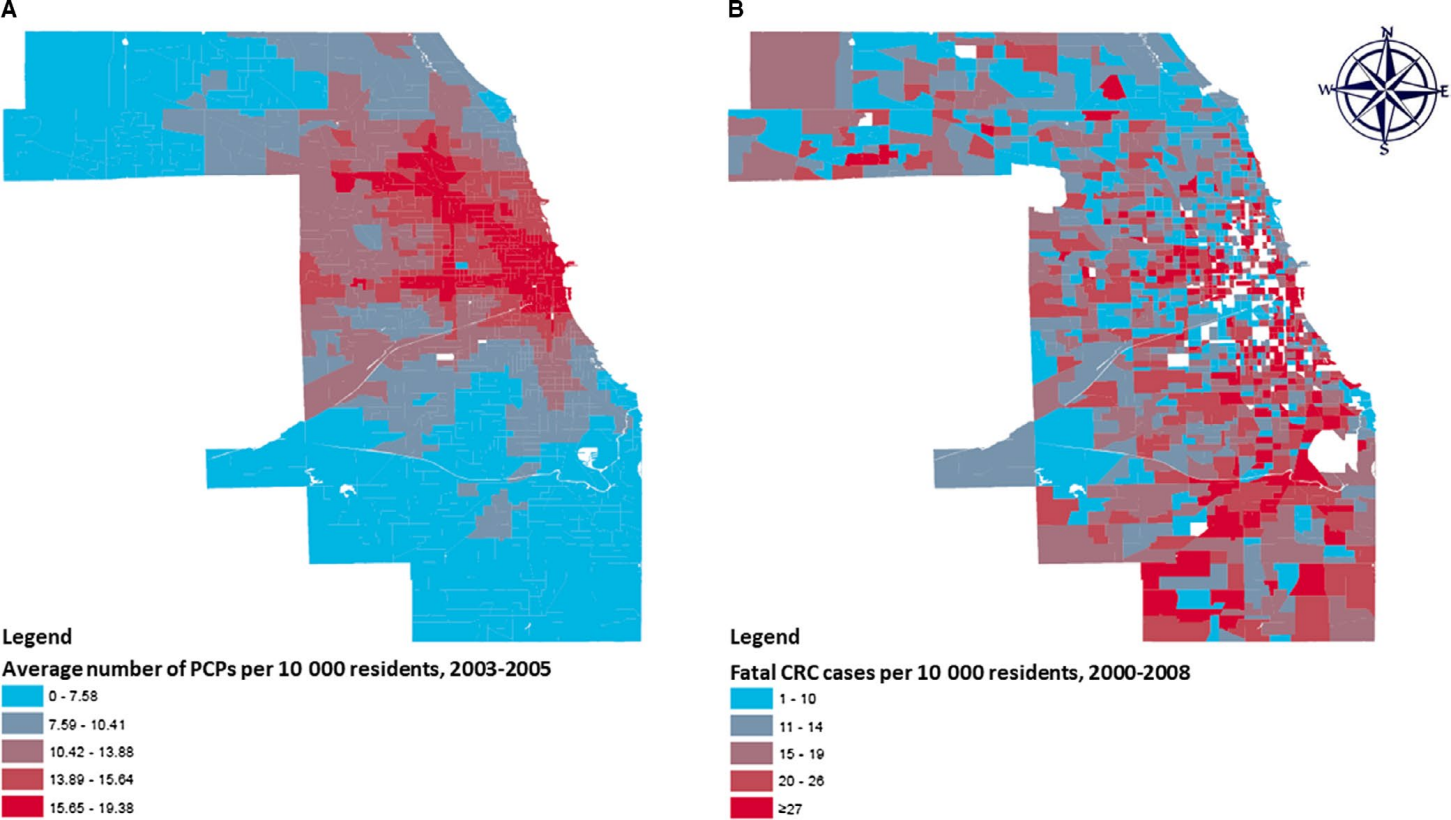
Mid‐period (2003‐2005) average number of primary care providers (PCPs) per 10,000 residents (panel A) and cumulative (2000‐2008) number of fatal colorectal cancer (CRC) cases per 10,000 residents (panel B) at the census tract level, Cook County, IL. Categories represent quintiles

Figure 2 shows the spatial distribution of average PCP access scores (expressed as PCPs per 10,000 residents) between 2003 and 2005 (panel A) and CRC‐specific cumulative mortality rates from 2000 thru 2008 (panel B) at the census tract level. Census tracts with access scores at or above the median quintile are concentrated near the center of the county and extend northwestward. Census tracts with access scores below the median are located mostly in the southern half of the county. The spatial distribution of CRC‐specific cumulative mortality rates is more complex. However, census tracts with CRC‐specific cumulative mortality rates at or above the median quintile also tend to be located in the southern half of the county.

Table 2 shows the association between spatial access to PCPs and CRC‐specific survival before and after sequential adjustment for various factors. Initially, census tract‐level PCP access score was not associated with disease‐specific survival (model 1). However, during sequential adjustments (models 2 through 5), increased tract‐level access to PCPs was associated with a lower risk of death from CRC (hazard ratio [HR], 95% confidence interval [CI] = 0.87 [0.79, 0.96], *P* = .008, highest vs lowest quintile; HR‐trend, 95% CI = 0.98 [0.96, 0.99], *P* = .048). Moreover, the association remained relatively constant. This was in contrast to the attenuation of the association between tract‐level concentrated disadvantage and increased risk of CRC death with sequential adjustments for case, tumor, and reporting facility‐level factors (models 3 through 5). Using subjects in highest quintile as the reference group, the lowest quintile of spatial accessibility was associated with a 1.14‐fold greater risk of death from CRC (HR, 95% CI = 1.14 [1.04, 1.26]).

**TABLE 2:** Hazard ratios for the association of census tract‐level access to primary care providers (PCP) at diagnosis with colorectal cancer (CRC)‐specific survival before and after sequential adjustment for various factors

Variables	Categories	Model 1^a^		Model 2		Model 3		Model 4		Model 5	
		HR	95% CI	HR	95% CI	HR	95% CI	HR	95% CI	HR	95% CI
Census tract‐level pcp access score^b^, ^c^	Q1 (lowest)	1.00	ref.	1.00	ref.	1.00	ref.	1.00	ref.	1.00	ref.
	Q2	0.96	(0.86, 1.06)	0.92	(0.84, 1.01)	0.90	(0.84, 0.97)	0.91	(0.83, 0.99)	0.91	(0.83, 0.99)
	Q3	0.99	(0.92, 1.10)	0.93	(0.84, 1.02)	0.92	(0.86, 0.99)	0.94	(0.87, 1.02)	0.94	(0.87, 1.02)
	Q4	1.00	(0.91, 1.11)	0.92	(0.84, 1.01)	0.94	(0.88, 1.02)	0.94	(0.87, 1.02)	0.94	(0.86, 1.03)
	Q5 (highest)	0.96	(0.87, 1.07)	0.85	(0.78, 0.94)	0.89	(0.83, 0.98)	0.88	(0.81, 0.96)	0.87	(0.79, 0.96)
Covariates:											
Census tract‐level SES											
Concentrated disadvantage	Q1 (lowest)			1.00	ref.	1.00	ref.	1.00	ref.	1.00	ref.
	Q2			1.11	(1.05, 1.17)	1.10	(1.02, 1.18)	1.09	(1.02, 1.18)	1.07	(0.99, 1.16)
	Q3			1.16	(1.07, 1.26)	1.14	(1.06, 1.23)	1.08	(1.00, 1.18)	1.06	(0.97, 1.16)
	Q4			1.21	(1.10, 1.33)	1.16	(1.08, 1.25)	1.11	(1.01, 1.21)	1.08	(0.98, 1.19)
	Q5 (highest)			1.40	(1.28, 1.53)	1.23	(1.13, 1.34)	1.16	(1.05, 1.29)	1.12	(1.00, 1.26)
Case demographics											
Race/ethnicity	White, non‐Hispanic					1.00	ref.	1.00	ref.	1.00	ref.
	Black, non‐Hispanic					1.29	(1.21, 1.37)	1.17	(1.09, 1.26)	1.16	(1.07, 1.25)
	Hispanic					0.95	(0.85, 1.06)	0.96	(0.86, 1.08)	0.94	(0.83, 1.05)
	Asian^d^					0.99	(0.81, 1.21)	1.01	(0.82, 1.25)	1.00	(0.81, 1.23)
	Other^e^					0.58	(0.47, 0.73)	0.68	(0.57, 0.83)	0.67	(0.56, 0.81)
Sex	Male					1.00	ref.	1.00	ref.	1.00	ref.
	Female					1.01	(0.96, 1.07)	1.02	(0.97, 1.08)	1.03	(0.97, 1.08)
Age, per y						1.01	(1.01, 1.02)	1.02	(1.01, 1.02)	1.02	(1.02, 1.02)
Tumor characteristics											
Primary anatomic subsite	Proximal colon							1.00	ref.	1.00	ref.
	distal colon							0.91	(0.87, 0.96)	0.91	(0.86, 0.95)
	Rectum							1.06	(1.01, 1.10)	1.05	(1.01, 1.10)
Stage	Local							1.00	ref.	1.00	ref.
	In situ							0.34	(0.28, 0.43)	0.34	(0.28, 0.43)
	Regional							2.75	(2.50, 3.04)	2.75	(2.50, 3.03)
	Distant							13.1	(11.6, 15.3)	13.3	(11.6, 15.2)
	Missing							4.65	(3.85, 5.60)	4.60	(3.83, 5.57)
	Unstaged							5.96	(4.19, 8.48)	5.87	(4.24, 8.12)
Reporting facility											
Safety net‐designated	No									1.00	ref.
	Yes									1.08	(0.98, 1.19)
Academic/university‐based^f^	No									1.00	ref.
	Yes									0.89	(0.82, 0.98)
Cook County CRC case volume	High (>= 75/y)									1.00	ref.
	Med (30 to 74/y)									1.01	(0.93, 1.11)
	Low (< 30/y)									1.12	(0.82, 1.24)

Abbreviations: CI, confidence interval; HR, hazard ratio; PCP, primary care physician; SES, socioeconomic status.

^a^ Model 1 adjusted for diagnosis year; models 2‐5 also adjusted for location of residence within Cook County at diagnosis (Chicago vs suburbs).

^b^ Median physician‐to‐population ratio by quintile: Q1, 4.2 per 10,000; Q2, 6.8 per 10,000; Q3, 9.2 per 10,000; Q4, 11.8 per 10,000; Q5, 15.6 per 10,000.

^c^ Number of CRC‐related deaths by quintile: Q1, 1704; Q2, 1573; Q3, 1623; Q4, 1666; Q5, 1438.

^d^ Asian and Asian‐Indian/Pakistani.

^e^ Pacific Islander, American Indian, Alaskan Native, other, and unknown.

^f^ Based on the American Association of Medical Colleges 1997 definition.

Table 3 shows the results of the “full” model (model 5 from Table 2) stratified by stage. Census tract‐level access to PCPs at diagnosis was associated with a lower risk of CRC death only among Cook County residents presenting with regional‐stage tumors (HR, 95% CI = 0.80 [0.69, 0.93], *P* = .004, highest vs lowest quintile; HR‐trend, 95% CI = 0.95 [0.92, 0.98], *P* = .009). An association of census tract‐level concentrated disadvantage with an increased risk of CRC was only observed in this stage group as well (HR, 95% CI = 1.29. [1.11, 1.49], *P* = .001, highest vs lowest quintile; HR‐trend, 95% CI = 1.05 [1.02, 1.08], *P* = .002).

**TABLE 3:** Multivariable Hazard Ratios for the Association of Census Tract‐Level Access to Primary Care Providers (PCP) at Diagnosis with Colorectal Cancer‐Specific Survival Stratified by Surveillance, Epidemiology, and End Results (SEER) Stage

Variables	Categories	SEER stage					
		Local (n = 9075)		Regional (n = 9056)		Distant (n = 7307)	
		HR^a^	95% CI	HR	95% CI	HR	95% CI
Census tract‐level PCP access score	Q1 (lowest)	1.00	ref.	1.00	ref.	1.00	ref.
	Q2	0.85	(0.68, 1.07)	0.96	(0.83, 1.08)	0.92	(0.84, 1.01)
	Q3	0.83	(0.64, 1.06)	0.86	(0.76, 0.98)	0.99	(0.89, 1.09)
	Q4	0.91	(0.70, 1.17)	0.93	(0.80, 1.09)	0.97	(0.88, 1.08)
	Q5 (highest)	0.83	(0.60, 1.15)	0.80	(0.69, 0.93)	0.96	(0.86, 1.07)
Covariates:							
Census tract‐level SES							
Concentrated disadvantage	Q1 (lowest)	1.00	ref.	1.00	ref.	1.00	ref.
	Q2	0.80	(0.62, 1.04)	1.13	(0.97, 1.33)	1.07	(0.96, 1.20)
	Q3	1.07	(0.86, 1.33)	1.10	(0.97, 1.25)	0.95	(0.87, 1.04)
	Q4	1.17	(0.96, 1.42)	1.16	(1.01, 1.34)	0.98	(0.86, 1.13)
	Q5 (highest)	0.97	(0.77, 1.21)	1.29	(1.11, 1.49)	1.00	(0.85, 1.18)
Case demographics							
Race/ethnicity	White, non‐Hispanic	1.00	ref.	1.00	ref.	1.00	ref.
	Black, non‐Hispanic	1.49	(1.21, 1.84)	1.21	(1.09, 1.34)	1.15	(1.05, 1.27)
	Hispanic	0.94	(0.72, 1.24)	1.04	(0.86, 1.25)	0.91	(0.75, 1.11)
	Asian^b^	0.71	(0.46, 1.09)	0.95	(0.67, 1.34)	1.06	(0.80, 1.42)
	Other^c^	0.71	(0.46, 1.12)	0.87	(0.66, 1.11)	0.62	(0.48, 0.82)

Abbreviations: CI, confidence interval; HR, hazard ratio; PCP, primary care physician.

^a^ Multivariable hazard ratios also adjusted for sex, age at diagnosis, primary anatomic subsite, reporting facility characteristics, location of residence within Cook County at diagnosis (Chicago vs suburbs), and diagnosis year.

^b^ Asian and Asian‐Indian/Pakistani.

^c^ Pacific Islander, American Indian, Alaskan Native, other, and unknown.

Finally, census tract‐level access PCPs in Cook County did not associate with CRC tumor stage at diagnosis or death from other causes (Tables S1 and S2, respectively). However, a consistent finding was the survival disparity between non‐Hispanic blacks and whites. Blacks were more likely than whites to die of their cancer overall (HR, 95% CI = 1.16 [1.08, 1.24], *P* = .0001) and across stages (HR, 95% CI range = 1.15 [1.05, 1.27], *P* = .005, for distant‐stage tumors to 1.49 [1.21, 1.84], *P* = .0002, for local‐stage tumors).

## 4 DISCUSSION

Access to primary care is an important facilitator of healthcare across the cancer continuum, from primary prevention through survivorship and beyond.^19^, ^20^ This study sought to establish whether spatial access to primary care physicians (PCPs) at the time of a CRC diagnosis is associated of cancer‐specific survival. Overall, the results indicate that increased spatial access to PCPs is associated with improved CRC‐specific survival, and that the positive “effect” appears to be independent of established prognostic factors such as age at diagnosis and race/ethnicity. However, the findings also indicate that any apparent favorable effect on CRC‐specific survival may depend on the stage of the cancer at diagnosis. In Cook County, IL, the positive association between spatial access to PCPs and survival was apparent only among residents diagnosed with regional‐stage tumors, and this was likely driving the association we observed in the population as a whole.

Spatial access to PCPs is a geographic indicator of potential access to primary healthcare services more broadly.^48^ In cancer, the focus has generally been on spatial access to screening and/or primary healthcare facilities and its impact on utilization of cancer‐related health services, stage at presentation and, less commonly, clinical course. The vast majority of research in this area involves breast cancer, and the results have been mixed.^49^ The most consistent finding has been a positive association between geographical access to mammography and increased utilization of mammography. Inverse associations with late‐stage breast cancer have also been observed, but this has been less consistent across studies.^21^, ^22^, ^23^, ^50^ Presently, data on the impact of spatial access to primary care on CRC outcomes are scarce. To the best of our knowledge, this is the first study conducted in US to observe an association between spatial access to PCPs and CRC‐specific survival.

The study findings are noteworthy for several reasons. First, they suggest that spatial accessibility to primary care, which is an organizational characteristic of the healthcare system, is a prognostic factor in colorectal cancer. This raises the possibility that it may also associate with the clinical outcomes of other common cancers such as those of the breast, lung, and prostate. Second, subjects in the lowest quintile of spatial access to PCPs had a 1.14‐fold greater risk of death from CRC compared to those in the highest quintile after sequential adjustment for other factors. This excess risk is similar in magnitude to that observed among non‐Hispanic blacks relative to non‐Hispanic whites (HR, 95% CI = 1.16 [1.07, 1.25]). Black race is a known and widely accepted risk factor for fatal CRC,^51^ and in our study population, residing in a census tract with relatively low spatial access to PCPs had a similar impact. Also, the lack of a dose‐response across the middle quintiles may reflect a threshold effect of spatial access to PCPs on CRC‐specific survival in our study population. Lastly, hazard ratios in the 0.80 to 0.87 range can be viewed as modest. However, we suspect that these may be conservative estimates of the association of PCP accessibility with CRC survival given the study setting. Cook County, IL is a predominantly urban and health services‐rich environment, and this can decrease the likelihood of detecting associations between spatial accessibility to care and health due.^15^ Nonetheless, an effect of spatial accessibility was still detected after controlling for multiple patient‐ and healthcare‐related factors that are known to effect CRC survival.

The design of this study did not allow for the identification of specific factors mediating the association of spatial access to PCPs and CRC‐specific survival. However, the observation that spatial access to PCPs was not associated with tumor stage suggest that factors during and after diagnosis and treatment play a role. Emery et al identify three “core themes” along the cancer continuum that are of high relevance to primary care providers, namely, risk assessment, care planning, and timely and accurate delivery of care.^19^ There is also increased appreciation of the central role that primary care providers play during the cancer survivorship phase, especially with regard to insuring a smooth transition from specialty oncology care back to primary care following active therapy.^20^, ^52^ Thus, we postulate that the measure of spatial access to primary care used in this study is an indicator of local health system infrastructure and services available to facilitate the planning, coordination, and delivery of cancer and cancer survivorship care.

This study has several limitations. We did not include physician practice locations in northwest Indiana, which is located along the southeast border of Cook County, IL. Information for case volume was available for the facility where the diagnosis was made but not at the primary care provider level. It is possible that providers weighted by case‐volume would affect our results; future research could develop a spatial access measure or model that incorporates a case‐volume estimate of each provider. Also, our analysis did not take health insurance status, provider referral patterns, and other aspatial access factors at the individual‐level into account. A geographic bias in survival that is directly related to PCP access is also possible if persons who are more likely to access PCPs to obtain a colonoscopy referral tend to reside near one another. It is clear that screening colonoscopy is an important tool in the prevention and early detection of CRC. However, in the case of CRC survival, there are several equally important factors, such as PCP referral for diagnostic testing among patients with symptoms, referral of newly diagnosed CRC for treatment, and optimization of survivorship with diet and lifestyle factors (among others) that cannot be independently accounted for through this study design. Lastly, the results and interpretations can vary depending on the size, number and configuration of the areal units studied. This problem is well‐known to geographers and spatial analysts as the modifiable areal unit problem (MAUP).^53^ Thus, the results of this study may be idiosyncratic to the setting and our choice of the census tract as the area unit of analysis; extrapolating the results to other settings must be done cautiously.

In conclusion, spatial access to primary care providers in this predominantly urban setting is a predictor of colorectal cancer‐specific survival independent of area‐level socioeconomic status, tumor stage, and other factors. Measures of spatial access to primary care likely reflect local health system infrastructure and services available for planning and delivering cancer‐related care. Future research is needed to determine which areas within the cancer care continuum are most affected by spatial accessibility to primary care such as referral for screening, accessibility of screening and diagnostic testing, referral for treatment, and access to appropriate survivorship‐related care.

## CONFLICTS OF INTEREST

None to disclose.

## AUTHOR CONTRIBUTIONS

Concept and design: Vincent L. Freeman, Sara L. McLafferty, Richard E. Barrett, Richard T. Campbell. Data acquisition: Vincent L. Freeman, Sara L. McLafferty, Benjamin J Booth, Richard E. Barrett. Statistical analysis: Oksana Pugach, Richard T. Campbell, Emma Boylan, Vincent L. Freeman. Interpretation of the data: Vincent L. Freeman, Keith B. Naylor, Emma E Boylan, Sara L. McLafferty, Oksana Pugach, Benjamin J Booth, Richard E. Barrett. Drafting of the article: Vincent L. Freeman, Keith B. Naylor, Emma E Boylan, Sara L. McLafferty, Benjamin J Booth. Critical article revision and final approval: Vincent L. Freeman, Keith B. Naylor, Emma E. Boylan, Sara L. McLafferty, Oksana Pugach, Benjamin J. Booth, Richard E. Barrett, Richard T. Campbell.

## Supporting information

Table S1

Table S2

Table S3

## Data Availability

The data used for this research are not shared due to statutory requirements of the State of Illinois.
