# Supplementary material for: Spatial access to primary care providers and colorectal cancer‐specific survival in Cook County, Illinois
Source: Cancer Med. 2020 Mar 4;9(9):3211–23. doi: 10.1002/cam4.2957 (PMC7196057; doi:10.1002/cam4.2957)
Supplement: Supplementary file 1 — Table S1 [file CAM4-9-3211-s001.docx]

| **Supplementary Table 1.** Multivariate Odds Ratios (OR) and 95% Confidence Intervals (CI) for the Association of Census Tract-Level Access to Primary Care Providers (PCP) with Surveillance, Epidemiology, and End Results (SEER) Colorectal Cancer (CRC) Stage at Diagnosis | | | | | | |
| --- | --- | --- | --- | --- | --- | --- |
| Census Tract-Level PCP Access Score  Quintile | SEER Stage | OR^a,b^ | 95% CI | |  |  |
|  |  |  |  |  | p-value | Interpretation: |
| Q2 (vs. Q1) | In Situ (vs. Local) | 1.05 | (0.90, 1.22) | | .54 | *Compared to residents in census tracts with access scores in the 1^st^ (lowest) quintile, the odds of residents of tracts in the 2^nd^ quintile being diagnosed with in situ stage CRC is 0.05-times higher than the odds begin diagnosed with local stage cancer, and so on.* |
| Q3 (vs. Q1) |  | 1.01 | (0.87, 1.18) | | .87 |  |
| Q4 (vs. Q1) |  | 0.93 | (0.79, 1.10) | | .41 |  |
| Q5 (vs. Q1) |  | 0.96 | (0.80, 1.15) | | .66 |  |
|  |  |  |  | |  |  |
| Q2 (vs. Q1) | Regional (vs. Local) | 0.95 | (0.87, 1.05) | | .33 | *Compared to residents in census tracts with access scores in the 1^st^ (lowest) quintile, the odds of residents of tracts in the 2^nd^ quintile being diagnosed with regional stage CRC is 0.05-times lower than the odds begin diagnosed with local stage cancer, and so on.* |
| Q3 (vs. Q1) |  | 0.96 | (0.87, 1.06) | | .45 |  |
| Q4 (vs. Q1) |  | 0.97 | (0.87, 1.07) | | .51 |  |
| Q5 (vs. Q1) |  | 0.92 | (0.82, 1.03) | | .15 |  |
|  |  |  |  |  |  |  |
| Q2 (vs. Q1) | Distant (vs. Local) | 0.92 | (0.82, 1.04) | | .17 | *Compared to residents in census tracts with access scores in the 1^st^ (lowest) quintile, the odds of residents of tracts in the 2^nd^ quintile being diagnosed with distant stage CRC is 0.08-times lower than the odds begin diagnosed with local stage cancer, and so on.* |
| Q3 (vs. Q1) |  | 0.93 | (0.82, 1.05) | | .22 |  |
| Q4 (vs. Q1) |  | 0.96 | (0.84, 1.08) | | .48 |  |
| Q5 (vs. Q1) |  | 0.95 | (0.83, 1.10) | | .50 |  |
|  |  |  |  |  |  |  |
| Q2 (vs. Q1) | Missing (vs. Local) | 0.77 | (0.58, 1.01) | | .06 | *Compared to residents in census tracts with access scores in the 1^st^ (lowest) quintile, the odds of residents of tracts in the 2^nd^ quintile with CRC cancer stage missing is 0.23-times lower than the odds begin diagnosed with local stage cancer, and so on.* |
| Q3 (vs. Q1) |  | 0.89 | (0.67, 1.17) | | .40 |  |
| Q4 (vs. Q1) |  | 0.84 | (0.62, 1.13) | | .25 |  |
| Q5 (vs. Q1) |  | 0.73 | (0.52, 1.03) | | .07 |  |
|  |  |  |  |  |  |  |
| Q2 (vs. Q1) | Unknown (vs. Local) | 0.88 | (0.69, 1.12) | | .30 | *Compared to residents in census tracts with access scores in the 1^st^ (lowest) quintile, the odds of residents of tracts in the 2^nd^ quintile being with CRC cancer stage unknown is 0.12-times lower than the odds begin diagnosed with local stage cancer, and so on.* |
| Q3 (vs. Q1) |  | 0.94 | (0.74, 1.20) | | .61 |  |
| Q4 (vs. Q1) |  | 0.83 | (0.64, 1.08) | | .16 |  |
| Q5 (vs. Q1) |  | 0.79 | (0.59, 1.06) | | .12 |  |
| ^a^Model derived using multinomial logistic regression.  ^b^OR adjusted for census tract-level SES, sex, age at diagnosis, race/ethnicity, primary anatomic subsite, reporting facility characteristics, location of residence within Cook County at diagnosis (Chicago vs. suburbs), and diagnosis year | | | | | | |
