# Supplementary material for: Spatial access to primary care providers and colorectal cancer‐specific survival in Cook County, Illinois
Source: Cancer Med. 2020 Mar 4;9(9):3211–23. doi: 10.1002/cam4.2957 (PMC7196057; doi:10.1002/cam4.2957)
Supplement: Supplementary file 2 — Table S2 [file CAM4-9-3211-s002.docx]

| **Supplementary Table 2.** Multivariate Hazard Ratios (HR) and 95% Confidence Intervals (CI) for the Association of Census Tract-Level Access to Primary Care Providers (PCP) at Diagnosis with Survival from Death due to Causes Other than Colorectal Cancer after Adjustment for Other Factors | | | | | | |
| --- | --- | --- | --- | --- | --- | --- |
| Variables |  | Categories | | HR^a^ | 95% CI | p-value |
| Census Tract-Level PCP Access Score^b^ | | | Q1 (lowest) | 1.00 | ref. |  |
|  |  |  | Q2 | 1.00 | (0.91, 1.10) | .99 |
|  |  |  | Q3 | 1.06 | (0.96, 1.17) | .26 |
|  |  |  | Q4 | 1.03 | (0.93, 1.13) | .59 |
|  |  |  | Q5 (highest) | 1.02 | (0.90, 1.16) | .75 |
| Covariates: | | | |  |  |  |
| *Census Tract-Level SES* | | | |  |  |  |
| Concentrated Disadvantage | | | Q1 (least) | 1.00 | ref. |  |
|  |  |  | Q2 | 1.01 | (0.92, 1.11 | .86 |
|  |  |  | Q3 | 1.00 | (0.90, 1.11) | .99 |
|  |  |  | Q4 | 1.11 | (1.01, 1.22) | .04 |
|  |  |  | Q5 (most) | 1.18 | (1.04, 1.34) | .009 |
| *Case Demographics* | | |  |  |  |  |
| Race/Ethnicity | | | Non-Hispanic White | 1.00 | ref. |  |
|  |  |  | Non-Hispanic Black | 1.05 | (0.97, 1.15) | .22 |
|  |  |  | Hispanic | 0.80 | (0.70, 0.92) | .001 |
|  |  |  | Asian^c^ | 0.69 | (0.53, 0.90) | .006 |
|  |  |  | Other^d^ | 0.47 | (0.34, 0.66) | <.0001 |
| Sex | | | Male | 1.00 | ref. |  |
|  |  |  | Female | 0.77 | (0.74, 0.81) | <.0001 |
| Age, per y | | |  | 1.06 | (1.05, 1.06) | <.0001 |
| *Tumor Characteristics* | | |  |  |  |  |
| Primary anatomic subsite | | | Proximal Colon | 1.00 | ref. |  |
|  |  |  | Distal Colon | 0.96 | (0.92, 1.01) | .13 |
|  |  |  | Rectum | 0.95 | (0.81, 0.98) | .012 |
| Stage | | | Local | 1.00 | ref. |  |
|  |  |  | In Situ | 1.05 | (0.95, 1.16) | .36 |
|  |  |  | Regional | 0.82 | (0.77, 0.88) | <.0001 |
|  |  |  | Distant | 0.58 | (0.52, 0.65) | <.0001 |
|  |  |  | Missing | 0.88 | (3.83, 5.57) | .11 |
|  |  |  | Un-staged | 0.83 | (4.24, 8.12) | .26 |
| *Reporting Facility* | | | |  |  |  |
| Safety Net-Designated | | | No | 1.00 | ref. |  |
|  |  |  | Yes | 1.04 | (0.91, 1.18) | .60 |
| Academic/University-Based^e^ | | | No | 1.00 | ref. |  |
|  |  |  | Yes | 0.90 | (0.82, 0.98) | .02 |
| Cook County CRC Case Volume | | | High (>= 75/y) | 1.00 | ref. |  |
|  |  |  | Med (30 to 74/y) | 1.04 | (0.96, 1.12) | .35 |
|  |  |  | Low (< 30/y) | 1.08 | (0.92, 1.27) | .35 |

Abbreviations: HR, Fine-Gray Subdistribution hazard ratio; CI, confidence interval, PCP, primary care physician; CRC,

colorectal cancer;

SES, socioeconomic status.

^a^HR; also adjusted for diagnosis year and location of residence within Cook County at diagnosis (Chicago vs. suburbs).

^b^Median physician-to-population ratio by quintile: Q1 = 4.2 per 10,000; Q2 = 6.8 per 10,000; Q3 = 9.2 per 10,000;

Q4 = 11.8 per 10,000; Q5 = 15.6 per 10,000

^c^Asian and Asian-Indian/Pakistani

^d^Pacific Islander, American Indian, Alaskan Native, other, and unknown.

^e^Based on the American Association of Medical Colleges 1997 definition
