# Supplementary material for: Spatial access to primary care providers and colorectal cancer‐specific survival in Cook County, Illinois
Source: Cancer Med. 2020 Mar 4;9(9):3211–23. doi: 10.1002/cam4.2957 (PMC7196057; doi:10.1002/cam4.2957)
Supplement: Supplementary file 3 — Table S3 [file CAM4-9-3211-s003.docx]

| **Supplementary Table 3.** Characteristics of cases included in the final analytic cohort (study population) vs. the characteristics of cases excluded | | | | | | | |
| --- | --- | --- | --- | --- | --- | --- | --- |
| Analytic cohort  (n=26,602) | | N | % | Excluded cases  (n=845) | N | % | P-value^a^ |
| Race/Ethnicity | White, non-Hispanic | 16866 | 63.4 | White, non-Hispanic | 479 | 56.7 | < 0.0001 |
|  | Black, non-Hispanic | 6934 | 26.1 | Black, non-Hispanic | 306 | 36.2 |  |
|  | Hispanic | 1708 | 6.4 | Hispanic | 45 | 5.3 |  |
|  | Other | 1094 | 4.1 | Other | 15 | 1.8 |  |
|  |  |  |  |  |  |  |  |
| Sex | Female | 13578 | 51 | Female | 457 | 54.1 | 0.082 |
|  | Male | 13024 | 49 | Male | 388 | 45.9 |  |
|  |  |  |  |  |  |  |  |
| Age at Diagnosis, y | < 45 | 1096 | 4.1 | < 45 | 69 | 8.2 | < 0.0001 |
|  | 45 to 54 | 2971 | 11.2 | 45 to 54 | 83 | 9.8 |  |
|  | 55 to 64 | 5098 | 19.2 | 55 to 64 | 97 | 11.5 |  |
|  | 65 to 74 | 6947 | 26.1 | 65 to 74 | 148 | 17.5 |  |
|  | >75 | 10490 | 39.4 | >75 | 448 | 53.0 |  |
|  |  |  |  |  |  |  |  |
| Stage | In Situ | 2255 | 8.5 | In Situ | 15 | 1.8 | < 0.0001 |
|  | Local | 9075 | 34.1 | Local | 109 | 12.9 |  |
|  | Regional | 9056 | 34 | Regional | 121 | 14.3 |  |
|  | Distant | 4827 | 18.2 | Distant | 75 | 8.9 |  |
|  | Unknown - Stage information missing | 572 | 2.2 | Unknown - Stage information missing | 11 | 1.3 |  |
|  | Unknown - Staging deferred or refused | 817 | 3.1 | Unknown - Staging deferred or refused | 514 | 60.8 |  |
|  |  |  |  |  |  |  |  |
| Vital Status | Alive | 12774 | 48 | Alive | 171 | 20.2 | < 0.0001 |
|  | Dead | 13828 | 52 | Dead | 674 | 79.8 |  |
|  |  |  |  |  |  |  |  |
| Cause of Death | Colorectal cancer | 8004 | 57.9 | Colorectal cancer | 496 | 73.6 | < 0.0001 |
|  | Other | 5824 | 42.1 | Other | 178 | 26.4 |  |

^a^Based on the chi-square test.
